# Contrasting effects of habitat discontinuity on three closely related fungivorous beetle species with diverging host‐use patterns and dispersal ability

**DOI:** 10.1002/ece3.4862

**Published:** 2019-02-16

**Authors:** Takuya Kobayashi, Teiji Sota

**Affiliations:** ^1^ Department of Zoology, Graduate School of Science Kyoto University Kyoto Japan

**Keywords:** Ciidae, dispersal, ecological specialization, landscape genetics, resistance‐based models

## Abstract

Understanding how landscape structure influences biodiversity patterns and ecological processes are essential in ecological research and conservation practices. Forest discontinuity is a primary driver affecting the population persistence and genetic structure of forest‐dwelling species. However, the actual impacts on populations are highly species‐specific. In this study, we tested whether dispersal capability and host specialization are associated with susceptibility to forest discontinuity using three closely related, sympatric fungivorous ciid beetle species (two host specialists, *Octotemnus assimilis* and *O*.* crassus*; one host generalist, *O*.* kawanabei*). Landscape genetic analyses and the estimation of effective migration surfaces (EEMS) method consistently demonstrated contrasting differences in the relationships between genetic structure and configuration of forest land cover. *Octotemnus assimilis*, one of the specialists with a presumably higher dispersal capability due to lower wing loading, lacked a definite spatial genetic structure in our study landscape. The remaining two species showed clear spatial genetic structure, but the results of landscape genetic analyses differed between the two species: while landscape resistance appeared to describe the spatial genetic structure of the specialist *O*.* crassus*, genetic differentiation of the generalist *O*.* kawanabei* was explained by geographic distance alone. This finding is consistent with the prediction that nonforest areas act more strongly as barriers between specialist populations. Our results suggest that differences in host range can influence the species‐specific resistance to habitat discontinuity among closely related species inhabiting the same landscape.

## 1 INTRODUCTION

Understanding how landscape structure influences biodiversity patterns and ecological processes are essential to ecological research and conservation practices. The extent and connectivity of local forests have a large impact on the species richness, abundance, and community structure of forest‐dwelling organisms (Hill et al., 2011; Laurance et al., 2002). Forest discontinuity is generally considered deleterious to the population persistence of forest‐dependent species, as discontinuity can limit gene flow across the landscape and reduce local population size. However, the actual impacts on populations can be highly species‐specific. While theoretical and empirical studies have reported negative effects of forest discontinuity on population persistence (Fahrig, 2002; Gibson et al., 2013), some species are less sensitive to forest discontinuity (Didham, Hammond, Lawton, Eggleton, & Stork, 1998; Lampila, Monkkonen, & Desrochers, 2005). This variation in sensitivity may be related to dispersal capacity and several ecological characteristics of the species (Henle, Davies, Kleyer, Margules, & Settele, 2004). Among ecological predictors of species sensitivity, specialization in habitat use and diet resources have been hypothesized to be key determinants (Keinath et al., 2017; Khimoun et al., 2016). Specialist species are less likely to disperse through areas where habitat patches are sparsely distributed, because, compared to generalists, they fulfill their resource requirements in smaller subsets of habitat patches and are more susceptible to local fluctuations of resources. Thus, nonforest areas act more strongly as barriers between specialist populations. In addition, specialist species tend to be patchily distributed, which increases differentiation among populations (Janz, Nylin, & Wahlberg, 2006) relative to generalist species. This pattern is expected to be more conspicuous in landscapes with discontinuous habitat. Correlations between ecological specialization and numerical responses of populations and communities to habitat fragmentation have been demonstrated in several taxa (e.g., birds: Devictor, Julliard, & Jiguet, 2008; butterflies: Steffan‐Dewenter & Tscharntke, 2000). However, such changes in population and community structure can be driven by several factors (e.g., environmental change accompanied by fragmentation, correlation between specialization, and movement behavior). Therefore, it is important to quantify the dispersal patterns of organisms in discontinuous habitats to improve our understanding of the effects of habitat discontinuity on population structure.

While the direct observation and quantification of movement behavior are costly and nearly impossible to conduct, the spatial genetic structure of a population enables us to infer the extent and routes of effective dispersal. Reduced dispersal between habitat patches will decrease gene flow among populations and thus increase genetic differentiation. Recent developments of landscape genetic methods allow researchers to test the effects of environmental change and habitat connectivity on gene flow between populations (Balkenhol, Cushman, Storfer, & Waits, 2015). In particular, a pairwise *F*_ST_ approach has been employed to test the effects of landscape quality on gene flow rates under different scenarios based on a null hypothesis of the absence of geographic structure (Balkenhol, Waits, & Dezzani, 2009). In this approach, an isolation‐by‐distance (IBD) scenario assumes that genetic differences increase with geographic distance due to limited dispersal across space, whereas an isolation‐by‐resistance (IBR) scenario predicts a relationship between genetic differentiation and resistance distance, indicating the differential effects of landscape features on dispersal (McRae, 2006). The IBR concept aims to characterize how genetic differentiation is shaped in heterogeneous landscapes, and “resistance” represents the cost to an organism to cross a particular environment, whereby a low resistance denotes ease of movement and a high resistance denotes restricted movement (Zeller, McGarigal, & Whiteley, 2012). When applying these scenarios to population responses to forest discontinuity, the IBD model indicates limited dispersal but the absence of impacts of habitat isolation, and the IBR model indicates significant effects of the loss of habitat continuity on population structure. Recently, a number of empirical landscape genetics studies have been conducted for a variety of taxa (Balbi et al., 2018; Beninde et al., 2016; Cleary, Waits, & Finegan, 2017; Crawford, Peterman, Kuhns, & Eggert, 2016; Frantz et al., 2012; Goldberg & Waits, 2010; Reid, Mladenoff, & Peery, 2017). However, most studies have focused on a single species or multiple species that largely differ in several characteristics (but see Engler, Balkenhol, Filz, Habel, & Rodder, 2014; Kelley, Farrell, & Mitton, 2000). Comparisons of closely related species that differ in their extent of ecological specialization on the same landscape would facilitate the examination of the effects of specialization on sensitivity to forest discontinuity.

Here, we perform a comparative population genetic study among closely related, sympatric ciid beetle (Coleoptera: Ciidae) species to test whether host specialization is associated with susceptibility to forest discontinuity. Ciid beetles are fungivorous and inhabit and feed on the basidiomes (fruiting‐bodies) of bracket fungi (Basidiomycetes). Most species of Ciidae feed on a relatively restricted number of fungal taxa (Fossli & Andersen, 1998; Lawrence, 1973; Økland, 1995; Orledge & Reynolds, 2005; Paviour‐Smith, 1960). Because their hosts, wood‐rotting bracket fungi, depend on the existence of dead woods, forests are considered potentially suitable and resource‐rich habitats for ciid beetles. The basidiomes of fungi are a relatively ephemeral and highly fluctuating resource, and they can occasionally disappear from small, isolated habitats. Fungus‐feeding species that can use multiple fungal species are expected to have a greater likelihood of fulfilling their resource requirements in such patches. Ciid beetles provide an ideal system for the study of spatial ecology in forest ecosystems, because they are abundant in number and depend on the basidiomes of bracket fungi at all stages of their life cycle. Several colonization experiments of insects on deadwood, including Ciidae, have suggested that the ability of insects to colonize isolated patches is highly species‐specific (Jonsell, Nordlander, & Jonsson, 1999; Komonen, 2008). Variation in colonization patterns may be driven by not only dispersal ability but also species‐specific ecological traits including host utilization. Our recent study demonstrated that host use differs even among three closely related species: *Octotemnus assimilis*, *O*.* crassus*, and *O*.* kawanabei* (Kobayashi & Sota, 2019). While five fungal species of *Trametes* and *Lenzites* are known to be main host species of *O*.* kawanabei*, *O*.* crassus* uses only two of them and *O*.* assimilis* uses the remaining three fungal species. Thus, *O*.* crassus* and *O*.* assimili*s are more specialized in host use than *O*.* kawanabei*. These closely related, sympatric species thus provide a unique opportunity to compare the effects of forest discontinuity on genetic structure among ecologically divergent species.

In this study, we compare population genetic structure among the above three *Octotemnus* species inhabiting the same landscape to test the prediction that ecologically specialized species (specialists; species with narrower host range) are more sensitive to forest discontinuity than generalist species (species with a broader host range). We hypothesized that nonforest areas will act more strongly as a barrier for specialist species than for generalist species and that compared to the simple IBD model, IBR scenarios will better explain the population structure of the specialist species when they do not differ in their dispersal abilities. We used microsatellite data and performed resistance surface optimization and applied the estimation of effective migration surfaces (EEMS) model to landscape population genetic structure of individual species. We evaluated the dispersal ability of focal species using morphological data. We found that different species showed varying levels of response to forest discontinuity, which can be explained by differences in dispersal ability and host specialization.

## 2 METHODS

### 2.1 Study species and sampling

**Figure 1:**
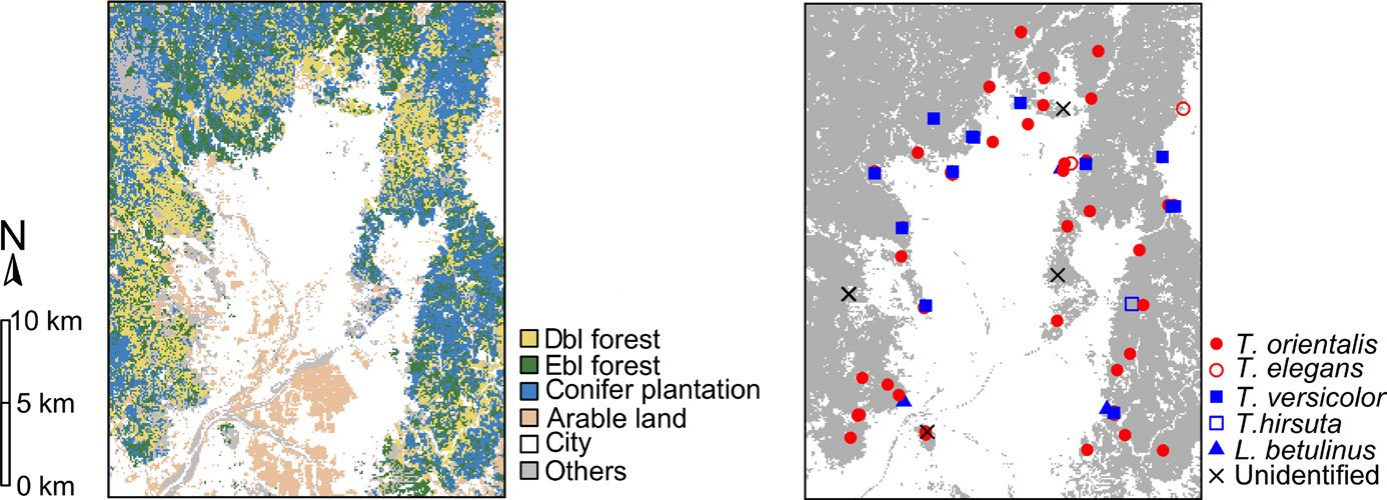
Maps of land cover and sampling sites in Kyoto (Japan). Left, land cover types in the study area: deciduous broad‐leaved forest (yellow), evergreen broad‐leaved forest (green), conifer plantation (blue), arable land (brown), city (white), and others (gray). Right, sampling points of host fungi. Symbols represent host‐fungal species from which beetles were collected. Areas of forest (gray) and nonforest (white; mainly city) are also shown

*Octotemnus crassus*, *O*.* kawanabei*, and *O*.* assimilis* are closely related species that exhibit different host‐use patterns (Kobayashi & Sota, 2019). The main host fungi of *O*.* crassus *are *Trametes orientalis* and *T*.* elegans*; the main host fungi of *O*.* kawanabei* are *T*.* orientalis*, *T*.* elegans*, *T*.* versicolor*, *T*.* hirsuta*, and *Lenzites betulinus*; and the main host fungi of *O*.* assimilis* are *T*.* versicolor*, *T*.* hirsuta*, and *L*.* betulinus*. The three ciid species are sympatric in the central part of Honshu Island. Sampling of *Octotemnus* species was conducted at 69 sites in Kyoto, Japan, from 2015 to 2018. The study sites were located in forests surrounding an unforested urban area. A land cover map from 1909 (available from the database of the Biodiversity Center of Japan: https://mapps.gsi.go.jp) indicates that the nonforest area has remained almost unchanged for 100 years. Therefore, the discontinuity among the study sites was considered to be longstanding. Insects were collected from the basidiomes of *Trametes* and *Lenzites* (and unidentified) species (Figure 1). All beetle specimens were preserved in 99% ethanol until DNA extraction. See Supporting Information Tables S1 and S2 for detailed information of specimens used in this study.

### 2.2 Microsatellite markers

New microsatellite markers were developed for the three *Octotemnus* species (*O. crassus*, *O. kawanabei,* and *O. assimilis*). For Illumina MiSeq next‐generation sequencing, genomic DNA was collected from a pool of 29–45 individuals for each species (Supporting Information Table S3). Genomic DNA was extracted using a DNeasy Blood and Tissue kit (Qiagen, Hilden, Germany). Genomic DNA was sheared in a volume of 50 µl using a Covaris M220 ultrasonicator (Covaris, Woburn, MA, USA). Then an Illumina paired‐end shotgun library was prepared following the standard Illumina TruSeq DNA Library Kit protocol with a targeted insert size of 550 bp (Illumina, San Diego, CA, USA). The generated library was validated using a Kapa Library Quantification kit (Kapabiosystem) and subsequently evaluated using the Agilent Technologies 2100 (Agilent Technologies, Santa Clara, CA, USA). Paired‐end sequencing was performed on the MiSeq Sequencer using a MiSeq reagent kit v3 (300 cycle). Overlapping paired reads were merged using PANDAseq (Masella, Bartram, Truszkowski, Brown, & Neufeld, 2012). The selection of merged reads containing microsatellites and the design of primers were conducted using QDD 3.1.2 (Meglecz et al., 2014). The universal tail sequence for fluorescent labeling of PCR fragments (Blacket, Robin, Good, Lee, & Miller, 2012) was added to forward primers. Loci were screened for PCR amplification success and polymorphism. Finally, 21 microsatellite loci were chosen for further characterization (Table 1).

**Table 1:** Characteristics of the newly developed microsatellite markers

Locus name	Motif	Forward primer	Reverse primer	Fluorescent	Size range	Number of alleles		
						*O. crassus*	*O. kawanabei*	*O. assimilis*
Olam01	AC	GTCGAACTCCTGCAACTGCT	GGCCTAATTCTTTCCCTGCT	FAM	112–126	1	6	7
Olam02	AG	ACCAGATGTTGGCATGATGA	AGTCAAGGACGGTGCAATTT	VIC	165–187	8	NA	NA
Olam11	AC	GTGATTTAAAGCGCTCTCCG	GTTTCTTCATCGAACCACGAACAAATG	NED	258–266	3	NA	NA
Olam13	AG	CATCATCCAGAGCAAACGAA	GTTTCTTCTTGCAGTCAGTTTGGGTGT	VIC	223–239	6	NA	NA
Olam14	AC	CCCGGTTGAAGATAATTCCA	GTTTCTTCGTGAGGACGCTTCACTGT	NED	132–140	4	1	NA
Olam16	AG	GTGACGGGTAGGAAACTTGC	GCTTTAAAGTTAATGTACCTGCTTTC	VIC	187–191	3	NA	NA
Olam17	AG	ATTTGTTATCGCTATCGGCG	GTTTCTTCGTTTGTCACTGACCGGAA	PET	218–236	5	8	10
Olam18	AG	CAAGAGCAGGCGTAAGTAAATTAAA	GTTTCTTTCCGTAATCCCAGACGACAT	FAM	158–190	6	12	6
Olam19	AG	ACACATTCGAGCAGATTTCG	GTTTCTTGTGCAGTTAGACCGCTCGTT	VIC	251–289	6	3	15
Olam27	AAGAG	TACACGAGACAGTGTGCCCT	TGTCAGAATCGCTCTTGTGG	NED	116–191	NA	11	NA
Olam31	AAT	GCGACTCTCGTTGCTAGGTG	GCGTCACCGTTTCACAATC	PET	182–188	NA	3	NA
Olam32	AAT	TCTCGTCTGCAACATTCTTCA	CGAAACGTAGAGAAGAGTAACCG	VIC	172–184	1	5	NA
Olam35	AAT	CATCTGTTTGATGATCAGTCCC	TACTCAAGCAGTGCACCCAG	NED	129–150	NA	8	NA
Olam39	AAT	GGGTGAAATTTGAGCACGA	TTTCCTATGGCCCAGTTTACA	PET	150–168	NA	NA	7
Olam43	AAT	TGAGCAACTACATTGCGACA	GCATCTAGGGTTGGAACGAC	PET	168–177	1	4	NA
Olam51	AG	TGCACCAACCAGTTATGCTG	GTTTCTTACATTATTCTCACAGAACGTCTTCG	FAM	158–184	NA	NA	12
Olam54	AG	TGTGAACGGGTTGAAATCTG	CGAGAGCGTCGCAAATCTAT	PET	222–260	1	1	16
Olam55	AC	CATTTCCAGAGCGTAATCAAG	CTATACATTCGGCGCGATTC	NED	254–282	NA	NA	13
Olam59	AG	TATGGCTTCCCATATCCTCG	CTAATGGATGCTCGCGATTC	VIC	253–309	NA	8	15
Olam60	AG	CTAAGAGGCGATATGTATTTCGAAGG	AAGAACAATCGAATCACGCC	VIC	199–207	NA	NA	5
Olam65	ACG	CAGTGAACCGGATGTGTACG	TCACCTTCGGTCTGTGTTTG	NED	206–236	6	NA	NA

Note

NA indicates that the locus was not amplified.

### 2.3 Genotyping and summary statistics

We amplified 9–10 microsatellite loci for each species with two multiplexes of five to six loci. Multiplex PCR was performed in 4.5 µl reaction volumes containing 1X Type‐it Multiplex PCR Master Mix (Qiagen, Hilden, Germany), 0.1 µM forward tailed primer, 0.2 µM reverse primer, and 0.2 µM fluorescent universal primer corresponding to the forward tailed primer. Cycling parameters consisted of the first step (denaturation, 95°C, 5 min), 28 cycles of the second step (denaturation, 95°C, 30 s; annealing, 58°C, 90 s; extension, 72°C, 75 s), and the third step (extension, 60°C, 30 min). PCR products were run on an ABI 3130XL capillary DNA analyzer (Applied Biosystems, Foster City, CA, USA) with the Gene Scan 500 LIZ size standard and then analyzed using the Peak Scanner software (Applied Biosystems).

In the following analysis, individuals collected from sites close to one another (typically <1 km), as well as those collected from the same fungal bodies, were treated as belonging to the same population. On average, four to six individuals per population were genotyped for each species. Populations with fewer than four individuals were excluded from the calculation of *G*″_ST_ (Meirmans & Hedrick, 2011). We checked null alleles using the Micro‐checker software (ver. 2.2.3; Van Oosterhout, Hutchinson, Wills, & Shipley, 2004), and examined departure from Hardy–Weinberg equilibrium (HWE) for populations with more than six individuals by exact tests implemented in GENEPOP (ver. 4.2; Rousset, 2008). Allelic richness for respective populations and population‐pairwise *G*″_ST_ were calculated using GenAlEx 6.503 (Peakall & Smouse, 2012). To visualize the population structure, a principal coordinates analysis (PCoA) was performed using GenAlEx. We conducted a linear mixed‐effects model with a maximum likelihood population effects parameterization (MLPE; Clarke, Rothery, & Raybould, 2002) using the *MLPE*.*lmm* function in R (R Core Development Team) to examine the effects of geographic distance on pairwise population genetic distance. The response variable was the genetic distance matrix, the fixed effect was the geographic distance matrix, and the random effect was population. The MLPE mixed‐effects parameterization accounts for nonindependence among the pairwise data.

### 2.4 Landscape genetics analyses

We conducted landscape resistance analyses to test our hypothesis that the species differ in their responses to landscape type (forest/nonforest). We obtained our land cover data from the database of the Biodiversity Center of Japan (http://www.biodic.go.jp/trialSystem/top_en.html). The land cover data of our study site are based on vegetation surveys conducted since 1999. The original vector format data were rasterized at 100‐m resolution (the smallest census unit of vegetation data) to perform subsequent landscape analyses. In addition, the original vegetation types were reclassified into two (forest and nonforest) or six (deciduous broad‐leaved forest, evergreen broad‐leaved forest, conifer plantation, arable land, city, and others) categories (Figure 1). Land cover types occupying <5% of the study area were reclassified as “other.” We followed the framework of optimization and selection of resistance surfaces using the “ResistanceGA” package (Peterman, 2018) in R. This method uses a genetic algorithm (GA; Scrucca, 2013) to optimize resistance surfaces to the pairwise genetic distances and conducts model selection to determine the best‐supported resistance surface. A linear mixed‐effects model with MLPE is fit to the data in model selection. We used pairwise *G*″_ST_ values between sampling sites as input data and assessed model fits using the Akaike information criterion (AIC). We assessed the relative support of three competing models: the IBD model, which proposes that gene flow is a function of the Euclidian distance among populations; the IBR model, which proposes that gene flow is a function of the resistance distance; and a null model (absence of geographic structure). Bootstrap analyses were conducted using the *resist*.*boot* function to evaluate the relative support of competing distance models. In each bootstrap replication, pairwise response and distance matrices are subsampled and fitted to the MLPE model to the data to obtain statistics. The percentage of instances of the IBD or IBR model being the best‐fit model was used as the support level.

### 2.5 Estimated effective migration surfaces

We visualized how the IBD relationship varies across geographic space using Estimated Effective Migration Surfaces software (EEMS; Petkova, Novembre, & Stephens, 2016). This method estimates effective migration rates based on genetic distances and then creates a visual representation of effective migration rates by interpolation. EEMS estimates the effective migration across space without the need to observe environmental variables and thus provides an exploratory tool for spatial population structure. This exploratory approach is complementary to the hypothesis‐driven resistance surface approach described above. We set the number of demes to 200 and ran three independent analyses with 1,000,000 burn‐in Markov chain Monte Carlo steps and 2,000,000 iterations. The results of three runs were combined using the rEEMSplots R package (Petkova et al., 2016).

### 2.6 Estimation of potential flight capability

**Figure 2:**
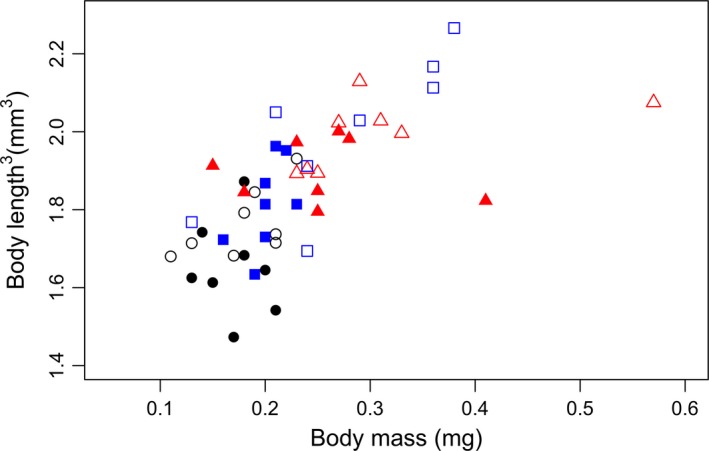
The relationship between body weight and body length. *Octotemnus assimilis*, *O. crassus*, and *O. kawanabei* are represented by black circles, blue squares, and red triangles, respectively. Open and solid shapes represent male and female individuals, respectively

It is believed that the study beetle species usually disperse by flight, because they have well‐developed hind wings and are frequently collected by flight‐intercept traps. We compared flight morphology of three species to evaluate relative dispersal ability. Specimens were collected from host fungi within the study site of landscape genetic analyses below (see Supporting Information Table S1 in detail). Beetles were killed and preserved in 100% ethanol for at least 48 hr and dried at room temperature for 24 hr. Body mass was measured using a digital balance (Sartorius BP 210D, Göttingen, Germany) to the nearest 0.01 mg. Subsequently measured beetles were digested in Nuclei Lysis Solution (Promega, Madison, WI, USA) with proteinase K (×mg/ml) at 55°C overnight, to easily dissect the hind wings. The left wing was removed and mounted in drops of mounting medium (Euparal). The length and width of the pronotum and elytra and the length, width, and area of the hind wings were measured using a VW‐9000 microscope with a VW‐600C camera and VH‐Z 100R zoom lens (Keyence, Osaka, Japan). In total, 48 individuals (eight males and eight females of each species) were measured. Wing loading (body mass divided by wing area) and wing aspect ratio (wing length divided by wing width) for each individual were calculated. Body mass was highly variable among individuals (Figure 2), likely because of differences in sexual development and gut contents; therefore, body length (sum of pronotal and elytral length) was used as a proxy of body mass to avoid such confounding influences. Pairwise differences between sex and species were examined using *t* tests, and Bonferroni adjustments were applied to *p*‐values.

## 3 RESULTS

### 3.1 Genetic diversity and population differentiation

**Figure 3:**
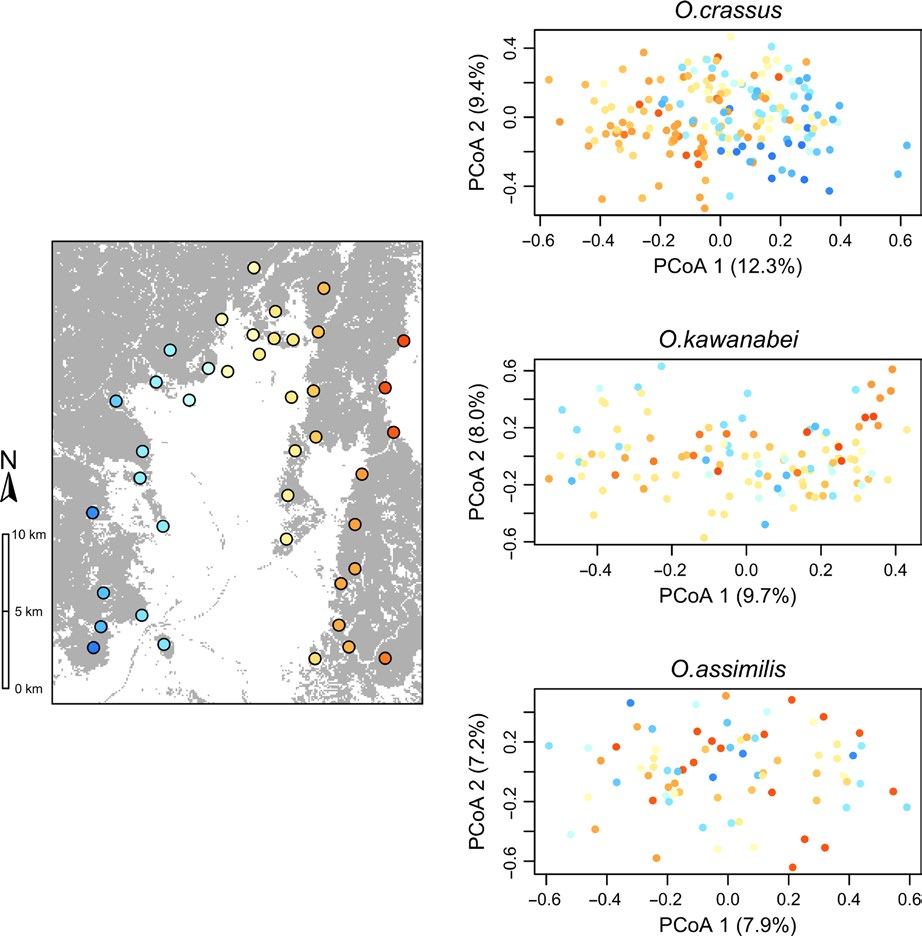
Principal coordinates analyses (PCoA) of microsatellite genotypes of individual beetles. Colors of dots correspond to those of the sampling sites shown on the map. Percentages indicated on axes indicate the amount of variance explained by PCoA1 and PCoA2

**Figure 4:**
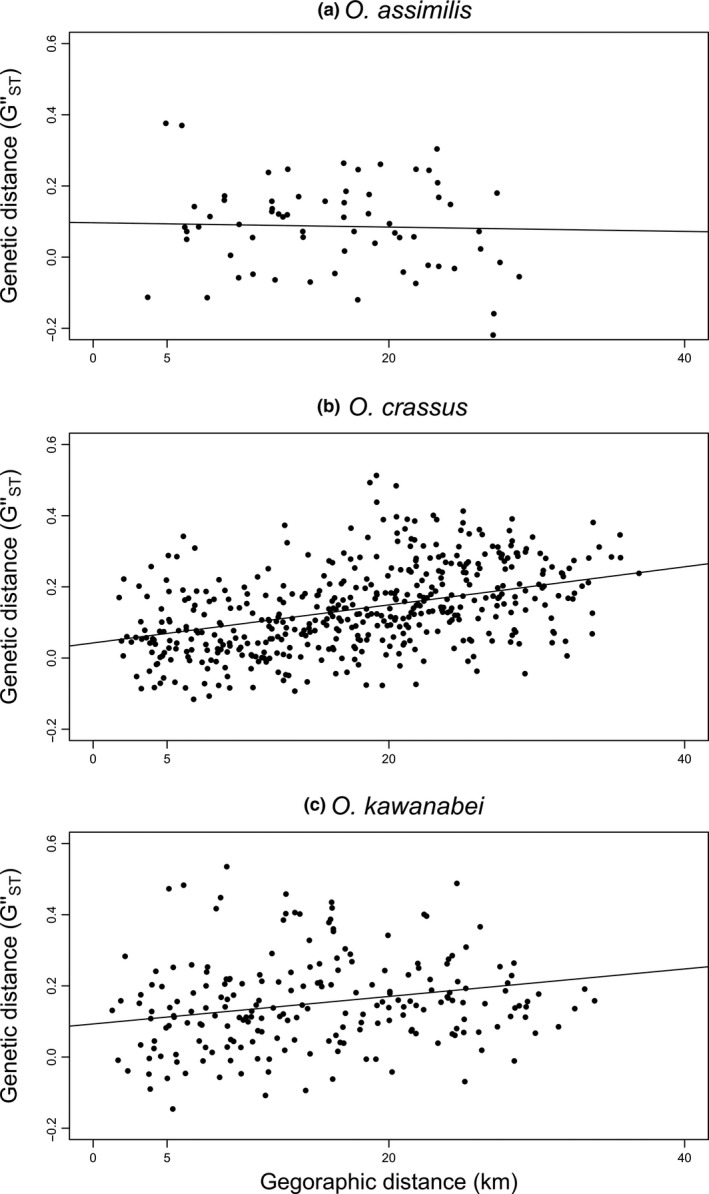
Relationships between pairwise *G*″_ST_ and geographic distance in (a) *Octotemnus assimilis*, (b) *O*.* crassus*, and (c) *O*.* kawanabei*. Regression lines are based on fitted values of linear mixed‐effects model with a maximum likelihood population effects parameterization (MLPE)

In total, 21 microsatellite loci were used for the three *Octotemnus* species in this study (Table 1). In all, 9, 10, and 10 loci were polymorphic across the sampled individuals of *O*.* crassus*, *O*.* kawanabei*, and *O*.* assimilis*, respectively. Although more than half of the markers were not shared among the three species, loci sets for the species exhibited similar allelic polymorphism. Deviations from Hardy–Weinberg expectations were observed in three, one, and four loci in *O. crassus*, *O. kawanabei*, and *O. assimilis*, respectively; in each species, only one population showed the deviation for each locus. In all species, we found significant genetic differentiation among sampled populations. Levels of differentiation ranged from weak in *O*.* assimilis* (global *F*_ST_ = 0.016, *p* = 0.017) to relatively high in *O*.* crassus* (global *F*_ST_ = 0.067, *p* < 0.001) and in *O*.* kawanabei* (global *F*_ST_ = 0.049, *p* < 0.001). Results of MLPE indicated that genetic distance and geographic distance were positively correlated in *O*.* crassus *(slope = 0.044, *t*‐value = 10.75, *n* = 465) and *O*.* kawanabei* (slope = 0.032, *t*‐value = 4.91, *n* = 210), but not in *O*. *assimilis* (slope = −0.003, *t*‐value = −0.26, *n* = 66; Figure 4). In addition, PCoA plots showed spatial genetic structure in *O. crassus *and *O. kawanabei*, in which genotypes of individuals differed between eastern and western sites; however, *O. assimilis* showed no appreciable spatial genetic structure (Figure 3).

### 3.2 Landscape resistance analyses

The model selection results differed among the three *Octotemnus* species, as did the optimized circuit resistance distance in *ResistanceGA* (Table 2). In *O*.* crassus*, the IBR model with six land cover categories was the best‐fit model, followed by the IBR model with two land cover categories. In the 6‐land cover IBR model, deciduous broad‐leaved forests and conifer plantations had lower resistance values (1 and 58, respectively), and evergreen broad‐leaved forests, arable land, and city had higher resistance values (1,212, 2,415, and 1,159, respectively). In the 2‐land cover IBR model, forests had a lower resistance value than nonforest land cover (1.0 vs. 13.6). The two IBR models were selected with a higher bootstrap percentage (65.4, 33.8%) than the distance model (1.3%), indicating effects of forest cover on population genetic structure. For *O*.* kawanabei*, the IBD model was supported, suggesting relatively limited dispersal; however, the estimated resistance values of forest and nonforest areas did not significantly differ (1.2 vs. 1.0 in the 2‐land cover model). We found no significant effects of IBD or IBR on genetic variation for *O*.* assimilis*.

**Table 2:** Model selection results of resistance surfaces for three *Octotemnus* species

	Model	*K*	AIC	AICc	*R* ^2^m	*R* ^2^c	LL	Percent.top
*O. assimilis (narrower host range)*	Null	1	−113.98	−117.58	0	0.44	59.99	NA
	Distance	2	−112.07	−114.73	0	0.43	60.03	NA
	Two land covers	3	−113.13	−112.13	0.07	0.46	60.57	NA
	Six land covers	7	−112.75	−78.75	0.06	0.45	60.38	NA
*O. crassus (narrower host range)*	Six land covers	7	−1,019.53	−1,008.66	0.59	0.91	513.77	65.4
	Two land covers	3	−1,006.63	−1,007.74	0.5	0.81	507.31	33.3
	Distance	2	−946.56	−950.13	0.18	0.41	477.28	1.3
	Null	1	−844.53	−848.39	0	0.27	425.27	NA
*O. kawanabei (broader host range)*	Distance	2	−446.32	−449.65	0.09	0.58	227.16	78.6
	Two land covers	3	−446.46	−447.05	0.1	0.6	227.23	18.6
	Null	1	−426.22	−430.01	0	0.51	216.11	NA
	Six land covers	7	−441.44	−426.83	0.23	0.66	224.72	2

Note

AIC: Akaike information criterion; AICc: adjusted Akaike information criterion; LL: log likelihood; *K*: number of parameters fit in each model; Percent.top: percentage of instances in which the distance (IBD) or land cover (IBR) model was the best‐fit model in bootstrap replications; *R* ^2^m and *R* ^2^c, the marginal and conditional *R* ^2^ values of the fitted MLPE model, respectively.

### 3.3 Estimated effective migration surfaces

**Figure 5:**
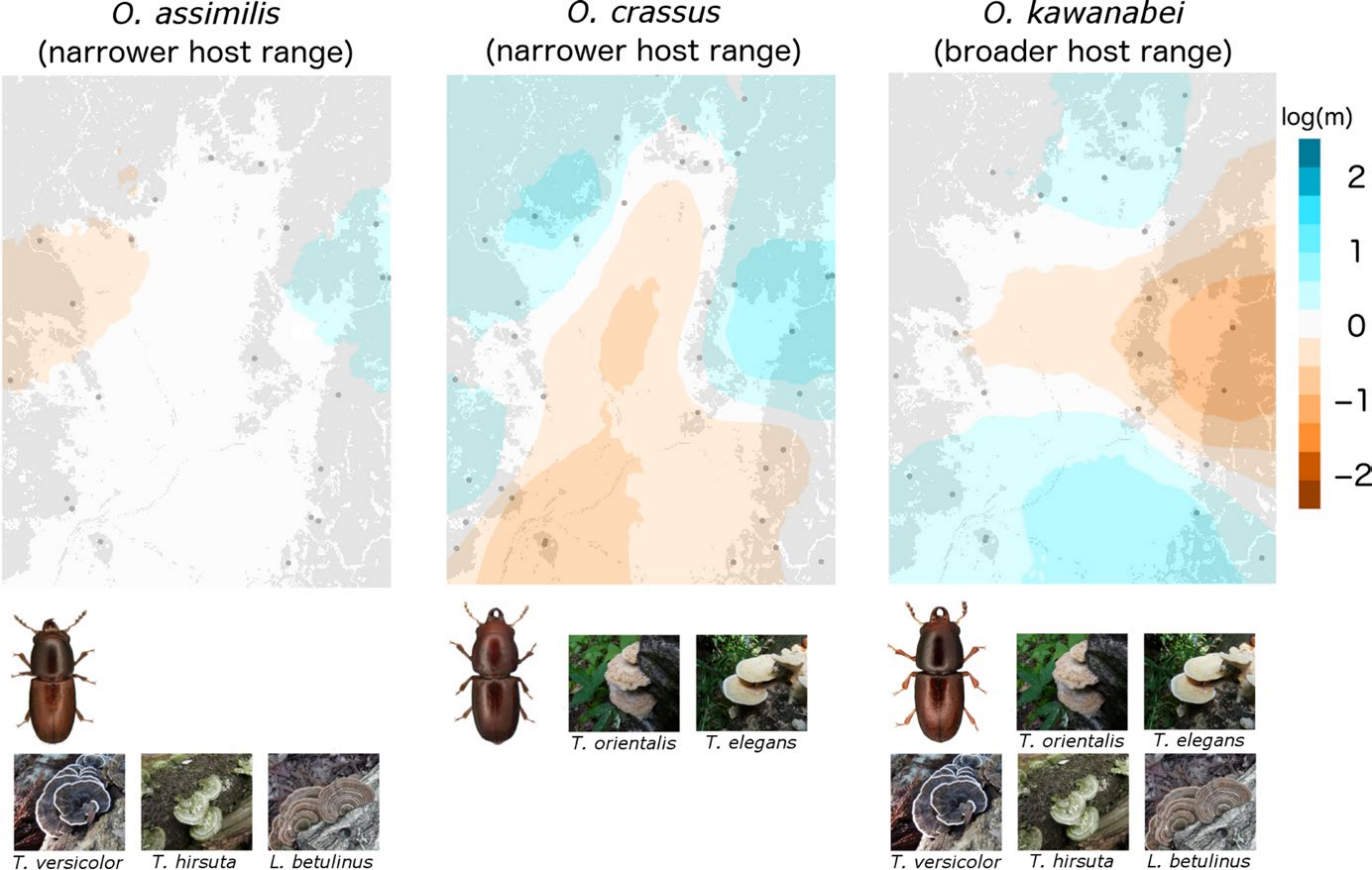
Estimated effective migration surfaces plot for *Octotemnus assimilis*, *O*.* crassus*, and *O*.* kawanabei*. Posterior mean migration rates m (on the log_10_ scale) are color‐coded. Blue areas indicate higher migration rates than those expected under isolation by distance (IBD), while the orange areas have lower migration rates than expected. Pictures of male specimens and host fungi for each species are shown beneath each map (photos from Kobayashi & Sota, 2019). Areas of forest (gray) and nonforest (white; mainly city) are also shown

Figure 5 presents the EEMS maps for each species. For *Octotemnus crassus*, the barriers of gene flow in the EEMS map (area with low estimated migration rate shown in orange) roughly correspond to the nonforest area. For *O*.* kawanabei*, a large barrier to gene flow separates the northern and southern areas of the study sites, but it does not correspond to forest land cover. For *O*.* assimilis*, the EEMS map shows a relatively homogeneous distribution of the effective migration rates.

### 3.4 Flight morphology

**Figure 6:**
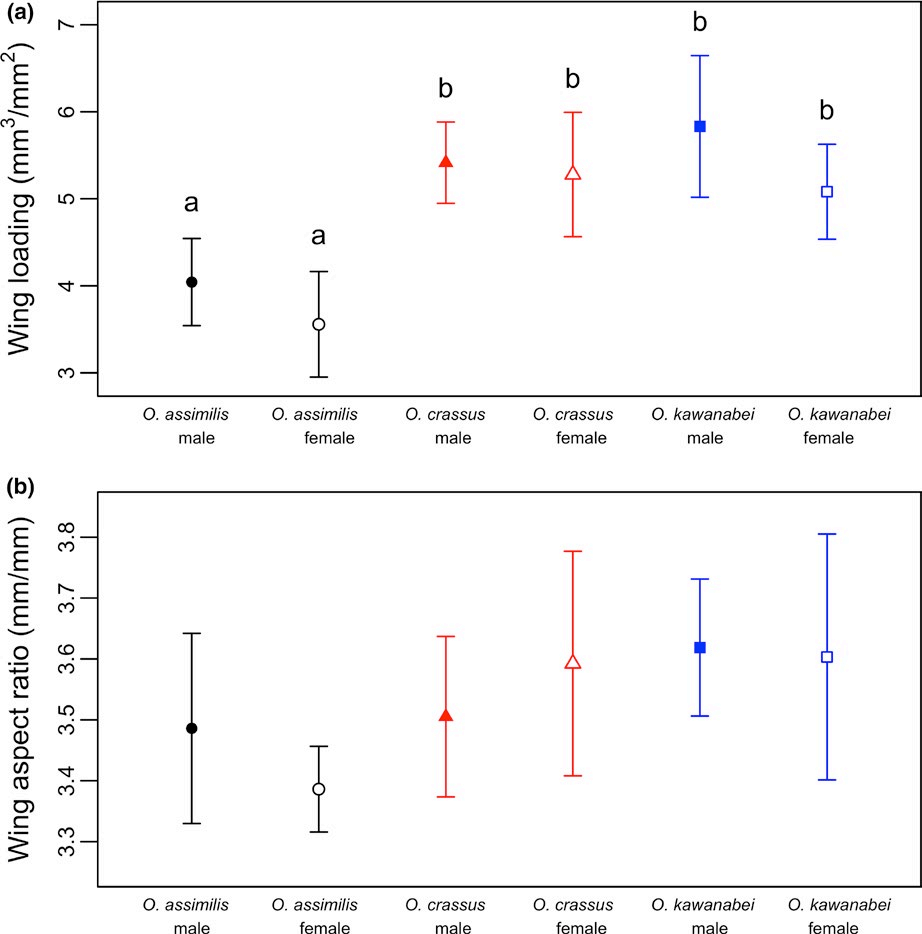
Measurements of (a) wing loading (cube of body length divided by wing area) and (b) wing aspect ratio (wing length divided by wing width) of the three *Octotemnus *species. Means with different letters are significantly different from each other (*t* test and Bonferroni adjustments). Error bars represent standard deviation

*Octotemnus assimilis* was smaller and lighter and had significantly lower wing loadings compared to *O*.* crassus* and *O*.* kawanabei* (Figure 6). On average, the wing loadings of both *O*.* crassus* and *O*.* kawanabei* were 1.41 times higher than that of *O*.* assimilis*. No significant differences were detected between sexes in any of the three species. Wing aspect ratios did not differ between species or sexes.

## 4 DISCUSSION

The closely related fungus‐feeding *Octotemnus* beetles exhibited differences in response to forest discontinuity. Analyses using both resistance surface and EEMS methods yielded similar results for each species. The interspecific differences could be associated with species’ differences in dispersal capability and ecological specialization. Among the three beetle species examined, *O*.* assimilis* presumably has higher dispersal capability than the other two species because its wing loading is much lower (Figure 6). The lack of spatial genetic structure on our study landscape for *O*.* assimilis* (Figures 4 and 3) was likely a result of the higher dispersal capability of this species. Thus, *O*.* assimilis* was unlikely to be affected by forest discontinuity despite its narrow host range, likely because high dispersal ability overcame the effects of host specialization.

The remaining two species, *O*.* crassus* and *O*.* kawanabei*, are similar in external morphology and wing loading, and hence potentially possess similar dispersal capabilities. However, the results of landscape genetic analyses differed between these two species: while support was detected for landscape resistance describing spatial genetic structure better than the IBD model in *O*.* crassus*, the genetic differentiation of *O*.* kawanabei* was described by geographic distance alone. This result suggests that the generalist *O*.* kawanabei* can disperse through nonforest areas better than the specialist *O*.* crassus*. The observed difference in sensitivity between species with similar dispersal ability suggests that host range is related to differences in sensitivity to forest discontinuity among closely related species inhabiting the same landscape. In addition, forest composition, and not just the difference between forest and nonforest categories, might be an important factor affecting the distribution of host fungi, given that the 6‐land cover IBR model had the best fit for *O. crassus*. The EEMS map of *O*.* kawanabei* indicates the reduction in gene flow between the northern and southern parts of the study area. This reduction in gene flow, however, does not correspond to the actual configuration of forest cover or landscape structure, which implies the presence of unknown barriers preventing the dispersal of individuals. Nonetheless, the overall results are consistent with the view that dispersal capability overrides the inhibitory effects of habitat isolation, and that when dispersal ability is low, ecological specialization can affect sensitivity to habitat isolation.

Our results are consistent with studies of the numerical response of specialists and generalists to habitat fragmentation in birds (Devictor et al., 2008) and butterflies (Steffan‐Dewenter & Tscharntke, 2000) with respect to the high sensitivity of specialists to habitat discontinuity. In addition, our results are also consistent with a recent study of the relationships between genetic differentiation and ecological specialization of birds in fragmented forests (Khimoun et al., 2016). However, our study is unique in several aspects. First, we used a comparative approach using closely related species. While analyses involving a large number of species provide insight into general patterns of organisms’ responses to landscape changes, it is difficult to examine the effects of particular characteristics because distantly related species differ in many traits. Comparisons of closely related species may provide better insights into the effects of key ecological traits that differ among species (e.g., host use). Second, we focused on the identity and number of host species, that is, ecologically important traits that are easy to define. Host choice is crucial for organisms, because hosts serve as primary resources of food and microhabitat for species that depend on them. Third, we evaluated the dispersal capability of focal species using morphological data, that is, separately from genetic data. A problem with investigating the relationships between species traits such as ecological specialization and response to habitat discontinuity is that these traits can correlate with dispersal capability (Jocque, Field, Brendonck, & De Meester, 2010). Therefore, understanding the direct effects of ecological specialization apart from dispersal capability on sensitivity to habit discontinuity requires an independent evaluation of the dispersal capability of focal species. Although numerous studies have examined ecological traits and the effects of landscape structure, few studies have evaluated the dispersal capability of study organisms. In this study, we used wing loading to evaluate each species’ capability of dispersal by flight. Flying is energetically more cost‐effective with lower wing loading (Angelo & Slansky, 1984; Arribas et al., 2012; Berwaerts, Van Dyck, & Aerts, 2002). Wing loading has been shown to affect flight performance in insects (Dudley & Srygley, 1994). For example, wing loading and flight distance of monarch butterflies are negatively correlated in flight‐mill experiments (Bradley & Altizer, 2005). However, it is uncertain whether wing loading is actually a reliable predictor of fight and dispersal capability in ciid beetles. Further laboratory and field studies are needed on the flight behavior of ciid species to clarify this matter.

Host use plays an essential role in the evolution and diversification of various organisms (Forbes et al., 2017; Hoberg & Klassen, 2002; Poulin & Morand, 2000). Specialization in host use can likely facilitate population differentiation and promote species diversification, because suitable habitats are generally more patchily distributed for specialists than for generalists and hence gene flow is more limited in specialist compared to generalist populations (Janz et al., 2006). This long‐standing hypothesis has been tested in a variety of taxa, and the results of many of these studies have agreed with the prediction (Brouat, Chevallier, Meusnier, Noblecourt, & Rasplus, 2004; Engler et al., 2014; Kelley et al., 2000; Zayed et al., 2005; but see e.g., Peterson & Denno, 1998). Our results from fungus‐feeding organisms are consistent with the prediction that specialization promotes population genetic subdivision.

A number of studies have explored the relationship between ecological specialization and sensitivity to habitat discontinuity; however, few studies have explicitly incorporated spatial genetic structure into the analyses. Such studies not only provide guidelines for conservation practices but also offer insight into the mechanisms of species diversification and biogeography. Recent developments of high‐throughput sequencers enable us to analyze many species and individuals in a single study at a low cost. By conducting additional comparative studies of multiple sets of closely related species, more generalized patterns can be explored.

## CONFLICT OF INTEREST

None declared.

## AUTHOR CONTRIBUTIONS

TK conceived the study, planned, and conducted all field and laboratory studies and data analyses, and wrote the paper. TS contributed to general ideas, sampling design, interpretation of data, and revising the manuscript. Both authors read the final manuscript.

## Data Availability

Raw sequence reads were deposited in the DDBJ Sequence Read Archive (DRA) under BioProject PRJDB6350 and BioSample Accession nos. SAMD00138639–SAMD00138641. Microsatellite genotypes and morphology measurement data were deposited in Dryad: https://doi.org/10.5061/dryad.6b16k45.
